# Comparison of Light-Based Devices in the Treatment of Meibomian Gland Dysfunction

**DOI:** 10.7759/cureus.41386

**Published:** 2023-07-05

**Authors:** Catarina Castro, João Heitor Marques, Ana Marta, Pedro Manuel Baptista, Diana José, Paulo Sousa, Pedro Menéres, Irene Barbosa

**Affiliations:** 1 Ophthalmology, Centro Hospitalar Universitário de Santo António, Porto, PRT

**Keywords:** ocular surface disease index, ocular surface disease, meibomian gland dysfunction, low-level light therapy, intense pulsed light

## Abstract

Purpose: To compare different light-based devices, namely, intense pulsed light (IPL) and IPL with low-level light therapy (LLLT), in the treatment of meibomian gland dysfunction (MGD).

Methods: This was a prospective, observational study that included patients with MGD. Group 1 included 58 eyes treated with IPL (eye-light®, Espansione Marketing S.p.A., Bologna, Italy), followed by LLLT (my-mask®, Espansione Marketing S.p.A., Bologna, Italy); Group 2 included 60 eyes treated with IPL (E>Eye®, E-Swin, Houdan, France); and Group 3 included 58 eyes treated with IPL (Thermaeye Plus®, OptiMed, Sydney, Australia). The presence of symptoms (Ocular Surface Disease Index (OSDI)) and ocular surface changes were evaluated at baseline, three weeks, and six months after treatment.

Results: At week three, there was an improvement in the OSDI in all groups (p<0.001), without differences among them (p=0.339). The lipid layer thickness (LLT) increased in Groups 1 and 2 (p<0.001), with a similar variation (p=0.144). Patients with superior OSDI and lower LLT at baseline had the greatest improvement in the respective parameters (p<0.001). The basal tear flow increased in Group 1 (p=0.012). Corneal staining (CS) significantly decreased in Groups 2 (p<0.001) and 3 (p<0.001). At six months, compared to three weeks, there was further improvement in the OSDI (p<0.001) and the LLT (p=0.007), in Group 1, and an increase in the presence of CS in Group 3 (p=0.011).

Conclusion: IPL treatment led to a sustained decrease in patients’ symptoms, even after six months. Different IPL devices seem to have different beneficial effects. Adding LLLT to IPL appears to have an additional long-term beneficial effect as well as positive effects on the lacrimal gland.

## Introduction

Meibomian gland dysfunction (MGD) is defined as a "chronic, diffuse abnormality of the meibomian glands, commonly characterized by terminal duct obstruction and/or qualitative or quantitative changes in the glandular secretion" [1]. Meibomian glands are found in the upper and lower eyelids, and they secrete meibum, a lipid-rich substance that contributes to the stability of the tear film and protects against its evaporation [2]. Hence, MGD may lead to ocular surface disease, with patients consequently experiencing dry eye symptoms [1].

The objective of MGD treatment is to improve the flow of meibomian gland secretion and, consequently, tear film stability. Over the years, many treatment options have become available. Treatment should start with environmental and dietary changes, and patients should also be advised to avoid contributing factors to dry eye disease. Furthermore, patients should be educated to perform eyelid hygiene, which includes eyelid warming and massage, which is a key element in the treatment of MGD. If necessary, topical medications can be added to the treatment, such as lipid-containing lubricants, antibiotics, and anti-inflammatory eye drops, as well as oral supplements with omega-3 fatty acids [1, 3]. When these treatments are not sufficient, other options can be considered.

Intense pulsed light (IPL) therapy utilizes a high-intensity non-laser light source, producing wavelengths ranging from 500 nm to 1200 nm. Many mechanisms could contribute to the improvement of the dry eye disease associated with MGD. IPL causes the obliteration of atypical erythematous blood vessels, reducing the reservoir of inflammatory mediators. It induces coagulation and necrosis of Demodex parasites, with a consequent decrease in the bacterial load on the eyelid. It also induces photobiomodulation, boosting cell proliferation, enhancing collagen synthesis, increasing local blood flow, and activating immunologic cells, among other actions. IPL influences the inflammatory cascade, leading to the upregulation of anti-inflammatory and/or downregulation of pro-inflammatory cytokines. Finally, IPL also induces heating of the meibomian glands and liquefaction of the meibum, unclogging the glands and allowing appropriate meibum secretion [4,5].

Low-level light therapy (LLLT), in turn, consists of using directional low-power, high-fluence, and monochromatic light in the red wavelength [6]. LLLT also induces photobiomodulation, which promotes cell damage repair, tissue regeneration, reduction of pain and inflammation, and prevention of tissue damage and, therefore, has a positive effect on MGD [7].

In recent years, some studies have shown a positive effect of IPL alone and in combination with LLLT in the treatment of MGD [8-10]. Despite this, evidence of the benefit of adding LLLT to IPL and comparisons between different IPL devices are lacking. Hence, our purpose was to compare different light-based therapy devices in the treatment of MGD.

## Materials and methods

Study design

This is a prospective observational study performed at the ophthalmology department of the Centro Hospitalar Universitário de Santo António, a tertiary care center in Porto, Portugal.

This study was performed in accordance with the tenets of the Declaration of Helsinki in its latest amendment (2013) and was approved by the local Institutional Review Board (Departamento de Ensino, Formação e Investigação, number 2021.143 (115-DEFI/118-CE)). All patients gave written informed consent.

Study population

Patients with a diagnosis of MGD, according to the International Workshop on Meibomian Gland Dysfunction [11], evaluated in the Dry Eye outpatient clinic of Centro Hospitalar Universitário do Porto, that were resistant to conventional treatment and aged over 18 years old, were considered for treatment. The exclusion criteria for treatment were: a loss area of the meibomian glands in the inferior eyelid (LAMG) superior to 40% (atrophic MGD), evaluated through infrared meibography with the IDRA® Ocular Surface Analyzer (SBM Sistemi, Inc., Torino, Italy); history of ocular trauma, intraocular surgery, or intraocular inflammatory disease in the previous six months; contact lens use in the previous six months; previous eyelid or lacrimal surgery; history of skin cancer anywhere; use of photosensitizing drugs; the presence of piercings in the treatment area; inability to comply with the treatment or the follow-up regimen. If one eye met the exclusion criteria, both eyes were excluded from the study.

Patients were allowed to use their usual artificial tears, but no changes were permitted after treatment was started. Patients were not allowed to use antibiotics or anti-inflammatory eye drops during the study period. Oral antibiotics and supplements were not allowed during the study period.

Only patients who completed all treatment sessions and attended all follow-up visits were included in the statistical analysis.

Treatment protocol

This study included 176 eyes from 88 patients: 58 eyes (29 patients) in Group 1, 60 eyes (30 patients) in Group 2, and 58 eyes (29 patients) in Group 3. Patients were randomly assigned to one treatment group.

Group 1: Included patients who underwent IPL and LLLT. IPL was performed with eye-light® with Optimal Power Energy® (Espansione Marketing S.p.A., Bologna, Italy) and LLLT with my-mask® (Espansione Marketing S.p.A., Bologna, Italy). For IPL treatment, protective eye shields were used. In each treatment session, the first three IPL pulses were placed along the inferior orbital rim with the device in the vertical position; the fourth pulse was delivered vertically behind the lateral canthus; and the fifth pulse was delivered with the device horizontally along the inferior orbital rim. The procedure was then repeated in the contralateral eye. The overall duration of the treatment was less than five minutes. The eye shields were then removed, and the LLLT was applied bilaterally through a red mask placed on the face for 15 minutes. LLLT treatment was performed without protective eye devices, but patients were instructed to close their eyes to ensure application to the upper and lower eyelids. The treatments were performed in three sessions, one week apart, in accordance with the manufacturer’s recommendations.

Group 2: Included patients who underwent IPL alone with E>Eye® (E-Swin, Houdan, France). In each treatment session, the first four IPL pulses were placed along the inferior orbital rim with the device in the vertical position, and the fifth pulse was delivered vertically behind the lateral canthus. The procedure was then repeated in the contralateral eye. Treatments were delivered over a layer of gel applied to the skin area to be treated which optimized cooling and light conduction, and protective eye shields were used. Patients were submitted to three treatment sessions on days zero, 15, and 45, respectively, as recommended by the manufacturer.

Group 3: Included patients who underwent IPL alone with Thermaeye Plus® (OptiMed, Sydney, Australia). In each treatment session, the first pulse was delivered horizontally, in the inferior orbital rim, followed by a vertical pulse, along the lateral canthus. The procedure was then repeated in the contralateral eye. After the first two pulses were applied to both eyes, the procedure was repeated, meaning that, in each session, four pulses were applied to each eye. Protective eye shields were placed, and the treatment was delivered over a layer of gel, as in Group 2. Patients underwent three treatment sessions on days zero, 15, and 45, respectively, as recommended by the manufacturer.

In all groups, before starting treatment, the patient’s face was cleaned of makeup or other skin products, and existing small, pigmented lesions were covered by protective adhesives. The level of energy delivered was set for each patient according to the degree of skin pigmentation, which was subjectively evaluated with the Fitzpatrick scale.

Patient evaluation

All patients were evaluated before treatment, three weeks after the last treatment session, and six months after the last treatment session.

All exams were performed in ascending order of invasiveness. Symptoms were evaluated with the Ocular Surface Disease Index (OSDI), a validated questionnaire [12]. Automated ocular surface analysis was performed with the IDRA® Ocular Surface Analyzer, which evaluated the non-invasive break-up time (NIBUT), the eye blink score (BS), the lipid layer thickness (LLT), the LAMG, and the tear meniscus height (TMH). The tear osmolarity was measured with the TearLab® Osmolarity System (Tearlab, San Diego, CA, USA). The basal tear flow was assessed with the basal secretion test (Schirmer strips, after the instillation of topical anesthetic). Lastly, slit lamp evaluation was performed, followed by the instillation of fluorescein dye to assess the presence of corneal fluorescein staining (CS).

Data analysis

In the presence of an OSDI score ≤12, the ocular surface was considered normal. Values between 13 and 22 indicated the presence of mild disease, between 23 and 32 indicated moderate disease and severe disease was considered in the presence of an OSDI score ≥33 [13].

Statistical analysis

Statistical analysis was performed using IBM® Statistical Package for Social Sciences (SPSS®) version 26 (IBM Corp., Armonk, New York, USA). The normality of the data was assessed using the Kolmogorov-Smirnov test. Descriptive statistics are shown as the mean ± standard deviation. For categorical variables, descriptive statistics are shown as absolute and relative frequencies. For evaluation of the changes over time in each group, repeated measures tests were performed for continuous variables and McNemar tests for categorical variables. For comparison between groups, a Kruskal-Wallis test was used in the presence of a skewed distribution, and a one-way analysis of variance (ANOVA) was used for variables with a normal distribution. To correct for baseline differences between groups, an analysis of covariance (ANCOVA) was performed. To prevent type I errors, post hoc corrections were performed, and a p-value inferior to 0.017 was considered statistically significant. Correlations were performed using the Pearson correlation coefficient (normally distributed data) and Spearman's rank correlation coefficient (data with a skewed distribution).

## Results

The participants' demographic data and baseline characteristics are detailed in Table 1.

**Table 1:** Baseline characteristics of the study group *Comparison between the three groups Note: statistically significant values are highlighted in bold (p<0.017) SD: standard deviation; OSDI: Ocular Surface Disease Index

	Group 1	Group 2	Group 3	p-value*
Age (mean±SD, years)	67.7±8.8	65.3±10.0	55.5±16.2	<0.001
Gender (female)	58.6%	80.0%	72.4%	0.036
Diabetes mellitus (%)	96.6%	66.7%	20.7%	<0.001
Phacoemulsification (%)	17.2%	43.3%	12.1%	<0.001
Artificial tears (%)	77.6%	76.7%	87.9%	0.231
Topical antihypertensive drugs (%)	20.7%	13.3%	3.4%	0.019

Regarding baseline ocular surface parameters, there was a statistically significant difference in the BS between Group 1 and Group 2 (p<0.001) and between Group 1 and Group 3 (p<0.001); the LLT between Group 1 and Group 2 (p<0.001) and between Group 1 and Group 3 (p<0.001); the osmolarity between Group 1 and Group 2 (p<0.001) and Group 1 and Group 3 (p<0.001); and the presence of CS between Group 1 and Group 2 (p=0.009). There were no other baseline differences in the studied parameters.

The OSDI score decreased in the three groups (p<0.001), three weeks after treatment. There were no differences in the OSDI score variation (p=0.339) between Group 1 (Δ=-23±18), Group 2 (Δ=-24±24) and Group 3 (Δ=-19±21). The variation of the OSDI score strongly correlated with the OSDI score before treatment in the three groups (Group 1: r(56)=-0.645, p<0.001; Group 2: r(58)=-0.718, p<0.001; Group 3: r(56)=-0.684, p<0.001). The LLT increased in Group 1 and Group 2 (p<0.001), three weeks after treatment. After correcting for the baseline difference in the LLT, there were no statistically significant differences in the LLT variation (p=0.144) between Group 1 (Δ=33±30 nm) and Group 2 (Δ=18±2 nm). The variation of the LLT strongly correlated with the LLT before treatment (Group 1: ρ(56)=-0.739, p<0.001; Group 2: ρ(58)=-0.501, p<0.001). Both the OSDI score and the LLT further improved in Group 1, six months after treatment (p= 0.007 and p=0.001, respectively).

In Group 1, there was a decrease in the TMH (p=0.004) and an increase in the osmolarity (p<0.001), in the BS (p=0.005), and in basal tear flow (p=0.012), three weeks after treatment. At six months, there was an increase in the LAMG (p=0.004) and osmolarity (p=0.001) and a decrease in the BS (p<0.001). In Group 2, there was a decrease in osmolarity (p=0.001) and in Group 3, there was a decrease in the NIBUT (p=0.007), three weeks after treatment. Changes in the ocular surface measurements and OSDI score in Group 1, Group 2, and Group 3 are detailed in Tables 2-4. 

**Table 2:** Changes in ocular surface parameters in Group 1 ^1^Baseline vs. Week 3 *Week 3 vs. Month 6 Notes: a) Statistically significant values are highlighted in bold. All values are summarized as mean ± standard deviation. b) A p-value inferior to 0.017 was considered statistically significant. When statistically significant changes were not found in the repeated measures test (represented in the “Change over time” column, no further comparisons were made between time points. OSDI: Ocular Surface Disease Index

	Change over time (p-value)	Before treatment	Week 3	p-value^1^	Month 6	p-value*
Non-invasive break-up time (s)	0.418	10.0±4.6	9.6±3.3	-	9.9±2.4	-
Eye blink score (%)	<0.001	89±22	99±4.2	0.005	63±17	<0.001
Lipid layer thickness (nm)	<0.001	47±25	80±21	<0.001	88±12	0.007
Loss area of the meibomian glands (%)	0.012	11±15	9±13	0.637	17±17	0.004
Tear meniscus height (mm)	0.014	0.33±0.16	0.29±0.17	0.004	0.32±0.26	0.340
Osmolarity (mOsm/L)	<0.001	298±9.7	306±12	<0.001	315±17	0.001
Basal secretion test (mm)	0.005	9.6±5.6	11.2±7.0	0.012	11.4±5.6	0.970
OSDI score	<0.001	44±22	22±17	<0.001	8±10	0.001

**Table 3:** Changes in ocular surface parameters in Group 2 ^1 ^Baseline vs. Week 3 *Week 3 vs. Month 6 Notes: a) Statistically significant values are highlighted in bold. All values are summarized as mean ± standard deviation. b) A p-value inferior to 0.017 was considered statistically significant. When statistically significant changes were not found in the repeated measures test (represented in the “Change over time” column, no further comparisons were made between time points. OSDI: Ocular Surface Disease Index

	Change over time (p-value)	Before treatment	Week 3	p-value^1^	Month 6	p-value*
Non-invasive break-up time (s)	0.609	9.8±3.7	9.8±2.3	-	9.5±2.4	-
Eye blink score (%)	0.049	71±23	60±15	-	61±14	-
Lipid layer thickness (nm)	<0.001	62±29	80±26	<0.001	84±17	0.091
Loss area of the meibomian glands (%)	0.277	12±11	9±7	-	10±11	-
Tear meniscus height (mm)	0.533	0.26±0.11	0.33±0.29	-	0.28±0.15	-
Osmolarity (mOsm/L)	0.008	315±20	305±14	0.001	307±17	0.967
Basal secretion test (mm)	0.068	9.9±6.2	11.5±7.7	-	11.3±5.5	-
OSDI score	<0.001	43±24	19±18	<0.001	24±21	0.078

**Table 4:** Changes in ocular surface parameters in Group 3 ^1^Baseline vs. Week 3 *Week 3 vs. Month 6 Notes: a) Statistically significant values are highlighted in bold. All values are summarized as mean ± standard deviation. b) A p-value inferior to 0.017 was considered statistically significant. When statistically significant changes were not found in the repeated measures test (represented in the “Change over time” column, no further comparisons were made between time points. OSDI: Ocular Surface Disease Index

	Change over time (p-value)	Before treatment	Week 3	p-value^1^	Month 6	p-value*
Non-invasive break-up time (s)	<0.001	10.7±2.9	9.2±2.8	0.007	8.5±2.0	0.070
Eye blink score (%)	0.373	64±17	60±56	-	61±16	-
Lipid layer thickness (nm)	0.030	74±26	85±18	-	81±22	-
Loss area of the meibomian glands (%)	0.831	11±10	12±11	-	12±10	-
Tear meniscus height (mm)	0.001	0.32±0.19	0.28±0.19	0.055	0.29±0.23	0.544
Osmolarity (mOsm/L)	0.843	308±17	311±15	-	311±15	-
Basal secretion test (mm)	0.554	13.4±8.7	13.8±8.8	-	12.0±6.2	-
OSDI score	<0.001	44±25	24±19	<0.001	29±20	0.044

**Figure 1:**
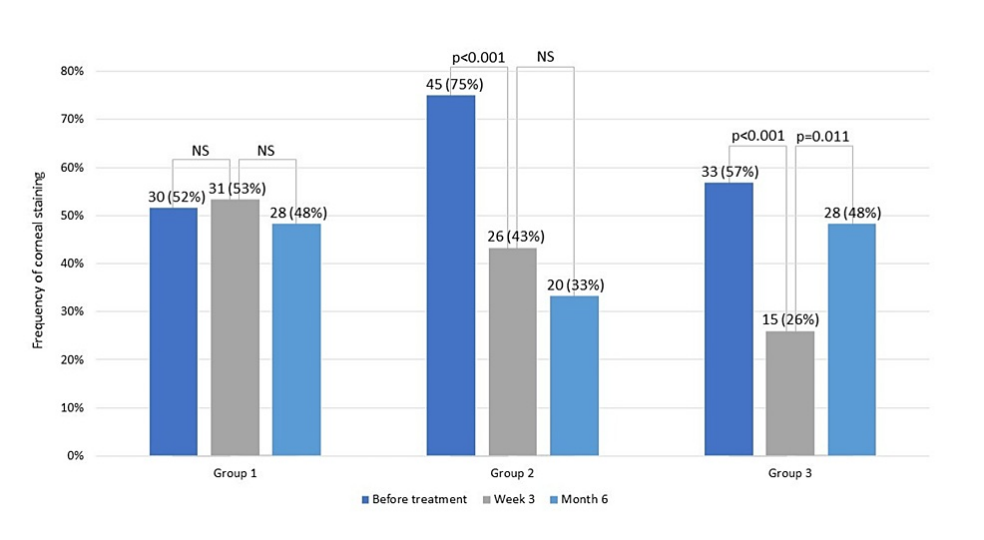
Presence of corneal fluorescein staining NS: not statistically significant Note: Values are represented as absolute and relative frequency

In Group 1, the presence of CS did not change over time (p>0.720). Four (14.3%) eyes developed de novo CS three weeks after treatment, and three (10.0%) eyes no longer had CS three weeks after treatment. Compared to week three, at month six, 14 (51.9%) eyes developed de novo CS and 17 (54.8%) eyes had resolution of the previous CS. In Group 2, there was a 42.3% decrease in the presence of CS at week three (p<0.001). Two eyes (13.3%) developed de novo CS three weeks after treatment, and 21 (46.7%) eyes that previously had CS no longer had it three weeks after treatment. Compared to week three, at month six, four (11.8%) eyes developed de novo CS, and 10 (38.5%) eyes had resolution of the CS. In Group 3, the presence of CS decreased by 54.5% (p<0.001) at week three, with a subsequent increase of 85.5% (p<0.011) at month six. Two (8.0%) eyes developed de novo CS three weeks after treatment, and 20 (60.6%) eyes had resolution of the CS. Six months after treatment, compared to week three, 18 (41.9%) eyes developed de novo CS, and five (33.3%) eyes had resolution of the CS. The frequency of CS at each time point is detailed in Figure 1.

No adverse events were reported during or after treatment in any group.

## Discussion

In our study, all devices led to a significant improvement in the OSDI score three weeks after treatment. Additionally, in Group 1 and Group 2, there was an increase in the outflow of meibomian glands, as indicated by the LLT improvement. Marques et al. [14] previously reported these changes. Improvement in LLT was not present in Group 3, indicating that Thermaeye Plus® may have a less beneficial effect on this parameter when compared to other IPL devices. In Group 1, six months after treatment, there was also further improvement in the LLT and in the OSDI score. Although the improvements in the OSDI score, in Groups 2 and 3, and in the LLT, in Group 2, were still present six months after treatment, indicating that the IPL’s benefit was sustained over time, only Group 1 had further improvements six months after treatment, which suggests that the LLLT continues to act for a longer period. Worse OSDI and LLT scores before treatment correlated with a greater improvement in these parameters after treatment, indicating that they are efficient even in patients with severe disease.

We also verified an improvement in basal tear secretion in Group 1 three weeks after treatment that remained present after six months. Marta et al. [15] hypothesized, in the preliminary results of this study, that this improvement could be due to the effect of LLLT rather than IPL since it was the only treatment applied directly to the upper eyelid. In our current study, we also verified that the improvement in basal tear secretion was not observed in both groups treated with ILP alone, which supports this theory. A previous study has shown that LLLT has a positive effect on the lacrimal glands of patients with dry eye disease, decreasing the presence of neutrophils and inflammatory cytokines [16].

In Group 1, there was a decrease in the TMH and an increase in the osmolarity. The decrease in TMH, despite being statistically significant, was still within normal values. On the other hand, osmolarity was normal before treatment, changing to abnormal values after three weeks and further worsening after six months. As suggested by Marta et al. [15], this may be due to a superior increase in the lipid content of the tear film when compared to the increase in the aqueous portion. However, this worsening of osmolarity did not translate into a worsening of patients’ symptoms. Moreover, although no significant changes were present at week three, there was an increase in the LAMG at month six in Group 1. Regardless, LAMG values also remained within normal limits. These changes were not observed with both IPL-only devices, which suggests they may be due to the LLLT action. This, combined with the fact that osmolarity further worsened six months after treatment, may be additional evidence that the LLLT effect has a longer action period.

Regarding CS, both groups treated with IPL alone had a significant decrease in the percentage of eyes with CS. In Group 2, despite not being statistically significant, there was a further decrease six months after treatment, with few eyes developing de novo CS, contrary to what happened in Group 3, in which there was an important increase in the presence of CS. Eyes submitted to IPL and LLLT did not seem to have substantial benefits regarding the presence of CS. As previously stated by Marta et al. [15], this group was mostly composed of diabetic patients, who seem to be susceptible to corneal changes, and this factor can have influenced our results [17]. Changes in the OSDI score did not reflect changes in CS. As we did not evaluate the degree of corneal damage, a possible explanation could be that CS that developed de novo may have been mild, thus not significantly increasing patients’ symptoms.

Few studies in the literature evaluate the effect of IPL alone or in combination with LLLT on the ocular surface. Arita et al. [18] compared the use of IPL in combination with meibomian gland expression with a control group that performed only meibomian gland expression. The group submitted to IPL showed superior improvement in the LLT, NIBUT, tear break-up time, lid margin abnormalities, CS, and meibum grade 24 and 32 weeks after starting treatment [18]. Piyacomn et al. [10] evaluated the effect of IPL treatment in comparison with a sham group. The authors reported an improvement in the tear break-up time in both groups, but in all visits other than baseline, the tear break-up time in the IPL group was higher than in the sham group. The OSDI score also improved in both groups, but only in the six-month visit was the OSDI score inferior in the IPL group compared to the sham group [10]. Wu et al. [19] compared two IPL devices, the E>Eye® and the M22 system® (Lumenis, Yokneam, Israel), finding that both led to improvements in the tear break-up time and meibomian gland secretory indexes. However, the M22 system® led to a superior improvement in meibomian gland secretory indexes, the first non-invasive break-up time, and the fluorescein break-up time [19]. Pérez-Silguero et al. [20] evaluated the long-term outcomes of IPL combined with LLLT. Three months after treatment, there was an improvement in the OSDI score, NIBUT, TMH, and osmolarity, followed by a worsening until the last follow-up. Despite this, at the last follow-up, all measurements still improved compared to the baseline [20]. Stonecipher et al. [8] evaluated the outcomes of IPL combined with LLLT in a single treatment in patients with MGD. As in our study, the authors verified an improvement in the OSDI score as well as in the proportion of patients with severe dry eye disease. There was also a significant improvement in the tear break-up time [8]. Solomos et al. [21] also evaluated the outcomes of IPL combined with LLLT. There was a significant improvement in the OSDI score, the tear break-up time, and the CS score. Contrary to our results, the basal tear secretion did not change after treatment [21]. To our best knowledge, only one study evaluated the use of LLLT alone in patients with dry eye disease [22]. The authors found no significant differences in tear break-up time, lid debris, lid swelling, lid telangiectasia, meibomian gland secretion, or expressibility scores between patients submitted to LLLT and the placebo group [22].

In our study, contrary to the previous studies discussed, we did not find a significant improvement in the NIBUT. However, in our study, the mean NIBUT value was already within the normal values. Regardless, direct comparisons with previous studies are difficult and should be made carefully, as studies used different devices, different treatment, and evaluation protocols, and had patients with different baseline characteristics, which can significantly influence the results.

Our study has some limitations, in particular the presence of baseline differences among groups, particularly age. Furthermore, patients were allowed to keep their usual artificial tears, with different active principles and administration regimens. Despite being told not to change the usage patterns and actively being asked about it, patients may not have reported the eventual changes performed. Another limitation of this study is that LLLT was added to a different IPL device than those used for treatment in Groups 2 or 3. Hence, we cannot know to what degree the IPL device itself contributed to the differences found in Group 1. We also did not evaluate the effect of LLLT alone, which would be important to understand the magnitude of its effect. However, the use of LLLT was commercially available with the eye-light®, and the use of IPL alone with this device was not recommended for the treatment of dry eye disease. We also did not have a control group, which would be important to quantify the placebo effect in the OSDI score. Regardless of these limitations, this is, to our best knowledge, the only study that presents a long-term comparison of eyes treated with IPL alone and in combination with LLLT. Furthermore, this is the only study that compares three different IPL devices.

## Conclusions

IPL treatment seems to lead to an improvement in patients’ symptoms that remains noticeable even after six months of treatment. Different IPL devices with different treatment protocols may lead to different beneficial effects. Adding LLLT to IPL seems to have an additional beneficial effect over time. MGD is complex and multifactorial, and the benefits of IPL and LLLT seem to go further than those evaluated in this study, explaining the improvement in the OSDI score in all groups, even when the objectivated changes were not similar. Further studies with even more comprehensive approaches, longer follow-up times, control groups, and larger samples will be necessary to help understand the effects of both IPL and LLLT, as well as the durability of the positive changes achieved with these treatments and the differences between devices.
